# Putting placebos to the test

**DOI:** 10.1371/journal.pbio.2001998

**Published:** 2017-02-21

**Authors:** Liza Gross

**Affiliations:** Public Library of Science, San Francisco, California, United States of America

In the summer of 1919, a slight but otherwise healthy 15-year-old English Navy recruit succumbed to epileptic fits so severe, his doctor wrote, “he threw five strong men about like kittens.” Navy medics exhausted options to treat the boy, and sent him home. His family doctor prescribed a testicular extract on the theory that the boy’s seizures could be related to his delayed sexual development. The doctor also allowed that some pharmacologists dismissed such extracts “as having only a psychotherapeutic value as placebos,” though he thought the effect was real. (Sex hormones play a complicated role in adolescent seizures, for the record [1].)

**Fig 1:**
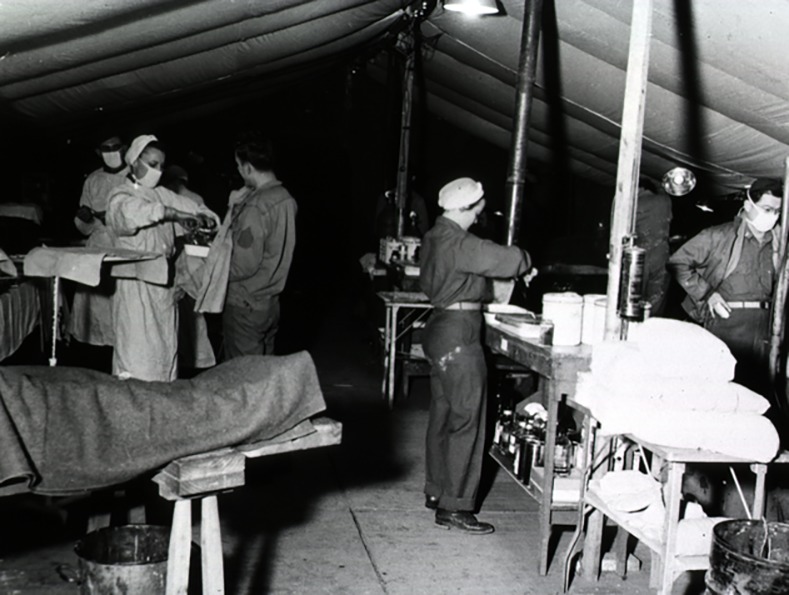
Expectations color one’s response to medical treatment. Henry Beecher, who treated soldiers like those in this surgery tent in Italy, was surprised to see a disconnect between the extent of a soldier’s wounds and his perception of pain. Beecher attributed their tolerance of pain to a psychological effect that counteracted sensations of pain. *Image credit*: *US National Library of Medicine*.

The pharmacologists likely embraced the definition of *placebo* offered a century earlier in Hooper’s Medical Dictionary as "any medicine adopted to please rather than to benefit the patient.” The curious power of the placebo received more scrutiny during the Second World War (Fig 1), when an American pharmacologist and anesthesiologist named Henry Beecher saw soldiers with shattered bones and shredded flesh rebuff his offers of morphine. A puzzled Beecher speculated that the men, so relieved to find themselves alive and safely removed from the horrors of battle, had entered a euphoric state that somehow overrode any feelings of pain, even after the ameliorative effects of shock had subsided.

Since then, numerous studies have described placebo responses, mostly in neurological disorders, including Parkinson’s, depression, chronic pain, and epilepsy. As Beecher suggested, expectations can trigger the response, which as a 2007 *PLOS Medicine* study found, tends to be greater in children, who are more suggestible [2]. Still, the biological basis of the placebo effect remains obscure, leaving many to question whether reported clinical gains reflect genuine improvements or artefacts of statistics or physiology.

Recent research in *PLOS Biology* [3] not only provides evidence that placebo treatment can produce significant pain relief but also flags a biomarker for the response. The placebo effect has been linked to neurobiological networks involved in expectations and inferences about what might happen based on past experience. So it stands to reason, the authors note, that the way these regions are wired in a person’s brain may influence their susceptibility to the response. To test this possibility, they scanned the brains of patients with chronic knee pain using functional magnetic resonance imaging (fMRI) before administering treatment in two small clinical trials. In the first, single-blind trial, patients were told they had a 50–50 chance of receiving a placebo or an active drug, though everyone received the placebo. The scans allowed the authors to identify brain differences between those patients who later responded to the placebo and those who did not.

In the second trial, patients underwent brain scans before being randomly assigned to receive a placebo or duloxetine, a drug used to treat chronic pain. (In this randomized double-blind trial, neither patients nor researchers knew who received which treatment.) The placebo-response biomarker identified in the first trial again emerged in scans of placebo responders in the second. The finding of the same biomarker indicates that brain biology can indeed predict a placebo response, which can be identified even before the placebo is given. Roughly half the patients who took the placebo in both trials reported feeling significantly less pain.

The authors also created a model to assess how a patient’s predicted placebo response might interact with the active drug. They found that for a third of the patients the drug may actually inhibit pain relief by blocking the placebo response.

“Given the enormous societal toll of chronic pain,” the authors write, “being able to predict placebo responders in a chronic pain population could both help the design of personalized medicine and enhance the success of clinical trials.” Knowing in advance that a patient might experience pain relief on a placebo, for example, suggests the placebo itself might be a useful treatment option. And the finding that FDA-approved pain medications might actually have an adverse effect by diminishing placebo-mediated relief suggests that clinical researchers should account for this possibility when designing pain treatment trials.

The study, like most studies of this phenomenon, does have limitations, including the small number of participants and the possibility that placebo-related improvements could be a function of spontaneous healing or symptom fluctuation.

Such limitations have long plagued researchers trying to disentangle the effects of a treatment drug from those of a placebo [4]. Randomized placebo-controlled double-blind trials are considered the gold standard to demonstrate a treatment’s safety and effectiveness, but using placebos in certain contexts and patient populations remains controversial, not least of all for the ethical questions raised [5]. The Helsinki principles for research on human subjects from the World Medical Association condone using a placebo when no proven or acceptable treatment exists. But ethicists and even researchers disagree over whether it’s appropriate to use a placebo if that means withholding a proven treatment from a patient in need.

The nature of the placebo used also raises ethical questions. Placebos can come in many forms of varying risk, from the sugar pill used in the *PLOS Biology* study to a surgical procedure that simulates a therapeutic intervention by, for example, drilling a hole in a patient’s skull without injecting a healing agent into the brain. A recent review of such “sham” surgery clinical trials in the *Annals of Medicine and Surgery* [6] found several confounding factors that could bias the interpretation of surgical placebo effects, prompting the authors to question the validity of sham surgery given the potential risks involved.

Researchers have devoted considerable attention to understanding how the doctor-patient relationship influences the placebo effect. A good or bad rapport can color a patient’s expectations, which can also be affected by suggestions or conditioned responses associated with a trial’s experimental design [7]. But even the best rapport can compromise a study if the investigators don’t fully understand the placebo effect (to the extent that anyone does). A recent survey of British surgical trainees in *BMC Surgery* [8] found that most respondents didn’t realize that the placebo effect produces real physiological changes, that it’s distinct from the natural history of the disease, or that conditioning can influence the response. Most also did not realize that the placebo effect can arise with any medical treatment.

Confusion over the use of placebos and their effects also crops up in surveys of patients. A small survey of patients and health professionals involved in clinical trials published in *PLOS ONE* [9] found that just two of twelve patients understood the goal of a placebo-controlled RCT, even after they’ve signed consent forms.

In a larger study published in *BMC Medical Ethics* [10], researchers reviewed 52 randomized placebo-controlled clinical trials in Finland and found that just 18 of the study protocols included a rationale for using a placebo. Only 12 bothered to include such rationales in participant information packets and only six informed patients of possible risks.

When Beecher found himself running out of pain killers while tending to U.S. soldiers on the Italian front, the story goes, he told the wounded soldiers that a saline-filled syringe held morphine. Many soldiers, thus deceived, were comforted. The ethical quicksand involved in deceiving patients was not lost on Beecher, though some have argued that the placebo effect is less a form of deception than a product of positive expectations [11]. Two decades later Beecher caused quite a stir when he called out his colleagues for ignoring ethical concerns in the name of scientific progress. Informed consent, he concluded, was among the most important ethical aspects of studies involving humans. Even though the challenges of adequately explaining a study’s aims and components meant that consent could sometimes be “exceedingly difficult to obtain,” he allowed, that was no excuse for giving up the effort.

Trying to explain what placebos are to patients and even to aspiring researchers, why they’re used in clinical research, and how they might work is clearly challenging. But few would argue that it’s not worth the effort.

For more detailed reading please see the associated PLOS Collection [12].
